# What Ethics for Bioart?

**DOI:** 10.1007/s11569-016-0253-6

**Published:** 2016-03-03

**Authors:** Nora S. Vaage

**Affiliations:** Centre for the Study of the Sciences and the Humanities (SVT), University of Bergen, Allégaten 34, Pb 7805, 5020 Bergen, Norway

**Keywords:** Bioethics, Art and morality, Bioart, Validation, Tissue culture and art project

## Abstract

Living artworks created with biotechnology raise a range of ethical questions, some of which are unprecedented, others well known from other contexts. These questions are often discussed within the framework of bioethics, the ethics of the life sciences. The basic concern of institutionalised bioethics is to develop and implement ethical guidelines for ethically responsible handling of living material in technological and scientific contexts. Notably, discussions of ethical issues in bioart do not refer to existing discourses on art and morality from the field of aesthetics. The latter framework is primarily concerned with how the moral value of an artwork affects its artistic value. The author argues that a successful integration of these two frameworks will make possible an ethics of bioart that is adequate to its subject matter and relevant for practice. Such an integrated approach can give increased depth to understandings of ethical issues in bioart, inspire new ways of thinking about ethics in relation to art in general and give novel impulses to bioethics and technology assessment. Artworks by the Tissue Culture and Art Project and their reception serve as the empirical starting point for connecting perspectives in art with those of bioethics, developing an ethics for bioart. The author suggests that consideration of the effect of these artworks is vital in validating ethically problematical applications of biotechnology for art. It is argued that the affective, visceral qualities of living artworks may spur the audience to adjust, revise or develop their personal ethical framework.

## Introduction

In the last three decades, biotechnological techniques and methods have increasingly been used for non-scientific and non-corporate purposes such as citizen science, biohacking, design and art. Today, hundreds of artists around the world use the different techniques of biotechnology, be it tissue culture, genetics or synthetic biology. These new media for art bring with them a whole new set of ethical issues not heretofore brought up in discussions of ethics in the context of art, as well as some that are unprecedented in discussions of the biotechnosciences.

Many artists explicitly seek to engage in the discussion of such issues. Sometimes, that very concern generates artworks that are themselves ethically problematic. This has led to a flourishing ethical discussion in relation to “bioart”. Bioart refers and may bring attention not just to biotechnology as a problematic but to wider biopolitical issues such as human beings’ relationships with other living things, human enhancement, the future of food production and the very notion of “technological fixes” to the “wicked” problems of our time. As such, it is part of an emerging range of technoscientific artefacts and activities—also including those of nanotechnology—that have a direct impact on society and speak to the direction in which we are collectively moving. Bioart, I will argue, can serve as a form of material technology assessment, but this does not exhaust its potential.

Discussions about bioart are often framed within the context of bioethics (see e.g. [42, 78]).^1^ This is understandable, since bioartworks are often presented as “responding to issues raised by biotechnology” ([43], p. 200),^2^ indicating that their interest lies primarily in how they relate to the life sciences and bioethical questions regarding, for instance, the proper use of living materials. William Myers has recently argued that the “tension between bioethics and technology is likely to underpin the most significant cultural developments of our age, and so the language of the life sciences—broadly speaking, and including its symbols, protocols, and objects—offers a rich communication tool for artists to use in probing our shifting ideas of identity” ([48], p. 14). However, bioethics is poorly suited for art-specific questions, which are also often posed in discussions of bioart. Whereas bioethical questions such as “how should we relate to other living beings” are indeed important aspects of many bioartworks, the reception of such art is dependent on the audience’s individual ideas of what art should do, and according to which parameters it should be judged.

This is further illustrated by the fact that other “fringe biotechnology” activities,^3^ although in practice often performed by some of the same actors for instance within community laboratories, are largely treated within a different ethical framework, emphasising biosafety and biosecurity (see e.g. [7, 25, 61]). Considering how closely interlinked they are as approaches to biotechnology, it is interesting to see how different discussions about the ethics of DIYbio and especially biohacking, with its connotations to “black-hat” computer hackers, are from discussions about bioart. Especially considering that the most publicised instance of a non-scientist being arrested on suspicion of bioterror intent is that of artist Steve Kurtz of the Critical Art Ensemble (see e.g. [46]), this is a striking example of how differently scholars and the general public deal with art, as opposed to other fringe biotechnology approaches.

In this paper, I argue that a richer understanding may be reached if we connect the ethical questions implicitly or explicitly raised by bioart to the question of what art can *do*, and more specifically how art is received.^4^ I propose, therefore, that insights from existing discussions of ethics in art can serve as tools for analysis of how one’s view of art will affect one’s response to bioethical questions posed in the context of bioart. Concurrently, art and morality discourses within aesthetics may benefit from the consideration of a new range of ethical issues. Since, as is argued by Myers [48] and Yetisen et al. [74], an increasing number of artists will be working in labs in the years to come, insight into ethical issues arising from such work is urgently needed.

The empirical focus in this paper is on the scholarly reception of artworks by Oron Catts, Ionat Zurr and their collaborators in the Tissue Culture and Art Project (TC&A).^5^ This choice is based partly on my case study at the SymbioticA Centre in Perth,^6^ where Catts and Zurr are based, and partly on the wide range of different responses generated by these artworks. After introducing the TC&A, I describe the categories of moralism, autonomism and contextualism, which have been identified as representing different approaches to art and morality, or, on a more fundamental level, to the role of art in society. These perspectives have not been extensively applied in relation to bioart. In the following section, I introduce the field of bioethics. Finally, I discuss whether and how the combination of these ethics can inform one another, taking into consideration a range of aspects of the aforementioned artworks and their reception in light of this new, interwoven framework.^7^ I will argue in favour of a contextualist position that considers each artwork in relation to its context, and in order to accentuate this point, I also draw on other artworks in the discussion.

While the bioethical questions often posed with regard to bioartworks are important, the affective impact they may have upon the viewer is perhaps even more important in relating to these pieces *as art*. Considering what art can *do*, I suggest that in the case of visceral, living artworks, the embodied response can induce reflection on the technologies in question and on our relationship to other living beings. This broaches the possibility that some bioartworks may contribute something to individuals’ ethical frameworks that might not be attained through other sources.

## Pig Wings and Extra Ears: Living Bioartworks

**Fig. 1:**
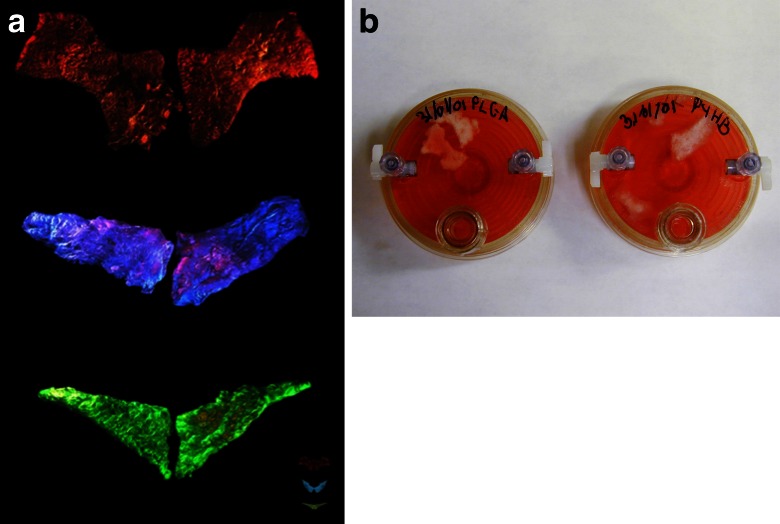
Tissue Culture and Art Project, *Pig Wings*, 2000–2001. Pig mesenchymal cells (bone marrow stem cells) grown over biodegradable polymers. **a** Photographed under coloured light: red bat wings, blue bird wings and green pterosaur wings. **b** The “bat” (*left*) and “bird” (*right*) wings in their culture vessels. Photo credits: Tissue Culture and Art Project. Reproduced with permission from the artists

Oron Catts and Ionat Zurr started working with biology in 1996, developing the TC&A Project based on the idea of using mammalian tissue culturing techniques to grow artworks. One of their early works, developed in collaboration with Guy Ben-Ary, as well as numerous scientific advisors including Joseph Vacanti, was the *Pig Wings* (2000–2002), three sets of “wings” grown from pig mesenchymal cells (bone marrow stem cells) on degradable biopolymers, in the shape of bird, bat and pterosaur wings. The piece played on how various creatures have been pictured with wings throughout history and how different types of wings indicated whether the figure was good (typically bird wings, as on angels and pegasuses) or bad/satanic (bat wings) (Fig. 1a). Another reference was the idiom “when pigs fly”, indicating something near impossible. With an ironic twist, the artists sought to show the limitations as well as the possibilities of current biotechnology [1]. The tissue sculptures, far from being actual flying pigs, measured only 4 × 2 × 0.5 cm each [18], and the artists state that they deliberately adopted an “aesthetics of disappointment”, counteracting the current hype of biotechnology (Fig. 1b).

For *Pig Wings* and other projects such as their *Semi-Living Worry Dolls* (2000), the TC&A engaged the audience explicitly in the *liveness* of their tissue-engineered artworks through their “feeding” and “killing” rituals. They “euthanized” the living artworks by exposing them to the non-sterile conditions of the world outside of petri dishes and incubators, allowing audience members to touch them with their (gloved) hands [15]. In contrast to the brightly coloured, large-scale photographic documentation of the *Pig Wings*, the (semi-)living, diminutive wings, consisting of stem cells subsisting in vitro without an immune system, shown in the gallery inside a rotating bioreactor contained in an incubator, were frail enough that contact with the outside world was in itself enough to kill them. The TC&A emphasised the necessity of consciously killing the cells themselves and allowing the audience to take part, “as there is usually no one who is willing to ‘adopt’ the semi-living entities” ([15], p. 239). In their killing rituals, although stressing their responsibility for their creations as well as elevating their “semi-living” creatures to a status of subjecthood, the artists also pointed out that “you might be killing more cells when you brush your teeth in the morning”.^8^

**Fig. 2:**
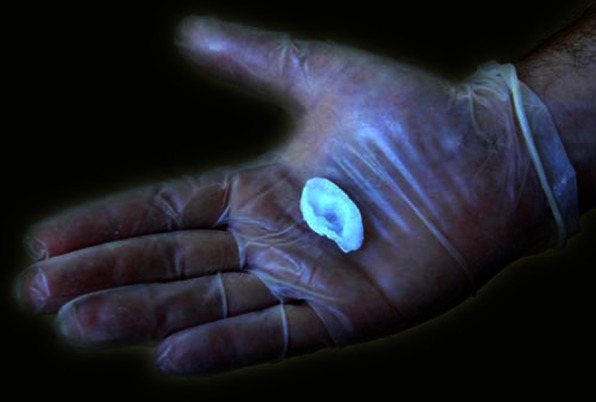
Tissue Culture and Art Project, *Extra Ear ¼ Size*, 2003. Photo credits: Tissue Culture and Art Project. Reproduced with permission from the artists

In 2003, Catts and Zurr collaborated with performance artist Stelarc to create an *Extra Ear ¼ Size* (Fig. 2), referencing the 1997 project in which Joseph Vacanti of Harvard Stem Cell Institute and his team had used tissue engineering to grow what looked like a human ear at the back of a mouse [9]. Using Stelarc’s left ear as a model, the artists grew an ear-shaped sculpture, but a quarter of the original size. While Stelarc’s intention was for the ear to be attached to his arm and made to emit sounds rather than receive them (he later had a full-sized ear surgically sculpted onto his left arm), exploring the phenomenon of excess in human enhancement [65], Catts and Zurr were interested in this body *part* as questioning “notions of the wholeness of the body” ([18], p. 27, see also [12]).

Since the early 2000s, Catts and collaborators such as Gary Cass and Stuart Hodgetts have also run a number of DIY biotech workshops, mainly for artists, but also for other interested parties, which again demonstrates that the boundaries between art and DIY are far more blurry in practice than in theoretical discussions. In 2006, TC&A developed the *DIY De-victimizer Kit Mark One* (*DIY DVK m1*), with the intention of extending “the life of parts of” deceased animals. The stated objective for this piece was to “allay some of the guilt people feel when they consume parts of dead animals” ([14], p. 18). Maciej Ożóg ([51], p. 51) observes that the death of the animals “becomes meaningful only in the context of the bad feelings felt by the perpetrators of the tragedy”, as this “re-life-ing” does nothing for the (already dead) animal in question.

Catts and Zurr have published extensively in academic journals about their artworks, their ironic intensions and how they speak to current developments in the biotechnosciences. They repeatedly stress their concern (see e.g. [16, 18]) with how life is increasingly seen as raw material to be manipulated, and explicitly seek to debunk what they call the “single engineering paradigm”, the “application of *real* engineering logic onto life” ([17], p. 28).^9^ Their artworks, on the other hand, are much more ambiguously presented. Some commentators have, however, deemed their approach ethically problematic. The following sections discuss different moral stances described within the ethical criticism of art and bioethics, which will aid our interpretation of how the two are connected in the reception of bioartworks.

## Moralism, Autonomism, Contextualism

The ethical importance of art has been discussed at least since the Ancient Greeks. Plato [52] was suspicious of the potential of poetry, painting and sculpture to sway people’s emotions, leading them away from the search for truth. Aristotle [3], on the other hand, emphasised the power of tragedy, in particular, to bring enlightenment through contemplation of an exemplary story. Although differing in their view of the value of art,^10^ they both evaluated it from what we would call a *moralist* point of view.

Moralists hold that art is subject to the same laws and norms as other activities in society. A moralist perceives the morality of art as having a direct impact on its aesthetic value. In other words: if an artwork is “morally defective”, it must be aesthetically flawed, too. The novel *Lolita* (1955) by Vladimir Nabokov is often mentioned as an example of the problem of moralism (see e.g. [11]). The formally exquisite prose of the book stands in stark contrast to its storyline about an unrepentant paedophile. A moralist would have to condemn it as artistically flawed, despite its aesthetical qualities. Similarly, Andres Serrano’s aesthetically striking, large-scale photograph *Piss Christ* (1987), which was created by submerging a plastic crucifix in a tank of the artist’s urine, has been met with charges of blasphemy, but has also received critical acclaim [62]. Moralists in the Platonic tradition view immoral art as dangerous because its aesthetic power might be seductive, whereas other moralists follow David Hume [36] in arguing that artworks with immoral contents will not be able to sway a morally conscious audience and will thus be aesthetic failures.

In the ethical criticism of art, moralism has long been considered an opposing tendency to *autonomism*,^11^ the view that ethical and aesthetic criticisms are separate. Moralism has traditionally been connected to the narrative and didactic power of art, whereas autonomism put more weight on formal aspects. Throughout the history of art, these two tendencies have existed side by side; now one taking precedence, now the other.^12^ The autonomist view can be found in the statement “art for art’s sake”, popular in Modernist art theory.^13^ The autonomy of art is directly connected to the idea of “artistic license”, that art should be free expression, unlimited by political and social conventions. R.W. Beardsmore [6] traced this idea back to Oscar Wilde’s demand that the critic ought to “recognize that the sphere of art and the sphere of ethics are absolutely distinct and separate” ([71], p. 191). An artwork can be ethically defect and still be aesthetically pleasing, and vice versa. Kieran Cashell points out that since autonomism does not acknowledge that works of art can validly possess ethical significance, it “is compelled to treat any works that do as hybrid deviations, as art mutations that cannot be considered purely artistic” ([11], p. 28). This is an inherently formalist view.

Autonomism comes up short when confronted with certain artworks whose moral and societal relevance is simply too great an aspect to be ignored. For instance, in *The Reincarnation of St Orlan* (1990–92) performance artist Orlan underwent a series of plastic surgeries to attain features from art historical models of beauty, including the brow of Leonardo’s *Mona Lisa*. The surgeries were staged as performances; Orlan was placed in a cruciform position, reading themed poetry during the procedure, which was filmed in its entirety. This project is an uncompromising confrontation with Western ideals of beauty, and as such, it may serve to discourage women from undergoing such surgical procedures [11]. A judgement of the artwork solely from an autonomist perspective (is the surgery performance and resulting facial and bodily features aesthetically interesting?) would miss the critical edge of this piece and, in the case of radical autonomism, would consider the work artistically poorer for containing such a politically charged message.

Paradoxically, moralists may sometimes be compelled to consider an artwork’s value in formalist terms. Daniel Jacobson, in “In Praise of Immoral Art” [37], emphasises how “the moralist”, when encountering “immoral” art, must either deny it any aesthetic value or continue somehow to accept it as art while remaining unmoved (or repulsed) by its offensive moral message. If the latter approach is chosen, what remains is a formalist judgement of the artwork separated from its content. Anthony Julius concludes that moralists and artists “cannot be reconciled, and that there is no third position available to harmonize the contrary perspectives” ([39], p. 9).

Noël Carroll’s [10] “moderate” moralism, however, hopes to achieve this third way. He suggests that moral value is not *always* relevant to the aesthetic value of the artwork but that morally defective contents may interfere with the audience’s appreciation of it. In other words, the moral value of a piece may in some cases directly influence its aesthetic value, which he defines as the degree to which they absorb us. The intention of the artist is an important factor to Carroll: if an artwork does not evoke a moral response *when one was intended by its producer*, the design of the work is faulty, and the work itself, therefore, is an aesthetic failure. But, following this logic, a work of art that was not intended to have a moral impact may well be aesthetically and artistically successful *without* arousing moral feelings in the viewer. In Carroll’s view, artworks that *do* engage our moral feelings may thus be evaluated “in terms of whether they deepen or pervert the moral understanding” ([10], p. 229). He argues that a moral artwork, when successful, can contribute to our moral education.

According to Jacobson’s “immoralist” view [27, 37], moral defects in art need not be aesthetical defects, even when relevant to the aesthetic judgement of the piece. They may actually *increase* its aesthetic value, rather than subtracting from it. Matthew Kieran argues “that morally defective imaginative experiences, including taking up attitudes and responding in ways that are morally problematic, are required to enable one more fully to understand things than one could otherwise have done” ([41], p. 63). This view finds common ground with moralism in contradicting autonomism’s insistence that morality should not be taken into account.

Both Jacobson and Carroll’s views are examples of “ethical pluralism”, a term that refers to any view acknowledging “that conflict between mutually opposed yet equally reasonable attitudes arises because moral values are neither exclusively oppositional nor commensurate with each other” ([11], p. 13, see also 10, 37). This relativist approach rests on the assumption that moral concepts do not apply equally to diverse situations. Another example of ethical pluralism is what Gaut [27] has called “contextualism”, the view that, *occasionally*, the unethical aspects of a morally questionable work may contribute positively to its artistic value. This term is seen as preferable in that it does not share immoralism’s implication that moral defects “are automatically aesthetic merits” ([11], p. 45).^14^ Rather, the “deployment of whatever principle may be required in the particular circumstances” should be our guide ([11], p. 46).

The above approaches showcase how the values of individuals influence their judgement of a work of art. What *I* see as the most important part may matter a lot less to *you*. How will these differing stances relate to bioart? Bioartists take widely different approaches, and their artworks, consequently, bring forth different ethical issues. On these grounds, I find a contextualist position to be the most productive perspective. A fundamental point is that these artworks should be treated *locally*, each artwork considered separately for its specific ethical relevance. In other words, the particular artwork’s artistic context, its geographical and historical situation, its relation to the methods used, as well as its political and societal dimensions, should be taken into account in the analysis. However, in discussions of ethical issues in bioart, a tendency of inferring from single artworks to “bioart” as such has so far been only too common (see e.g. [29, 67]).

Conceptually, a tradition that goes back at least to Plato has seen aesthetics and ethics as intimately intertwined, in the search for truth, beauty and goodness. However, in contemporary art, in contrast to the conventions of earlier times, the aim is rarely to give pleasure through the experience of harmonious beauty. Instead, artists seek to reflect some aspect of human existence, to provoke, critique or create immersive experiences. Although this can be done within the autonomist ideal, a large portion of contemporary artworks directly engage with issues in society, and most bioartworks fall within this sphere. Some are explicitly political and activist, for instance targeting genetically modified organisms (GMOs), as in Critical Art Ensemble’s *Transgenic Bacteria Release Machine*, which combined two public fears, GM and bacteria, and sought to inform the public about both. The audience were left to decide for themselves, based on the information they were given, whether they wished to release crippled non-pathogenic gut *Escherichia coli* bacteria, transformed with DNA fragments, into the environment (see e.g. [46]).

Ordinarily, we are made aware of our moral framework only when faced with difficult decisions, whether as individuals, as representing the interests of individuals (as is often the case for attorneys, next-of-kin, or GPs), or as a society (in which case politicians, various experts and NGOs tend to be key players). It can be argued that experiencing art can create an opportunity to critically examine or develop that moral framework. Although this need not be the raison d’être of the artwork, it can be an important factor for ethical validation.

While discussions of art and morality from aesthetics scholars can serve to qualify and explain some of the responses to bioart, there is a good reason why these artworks are often discussed from the perspective of bioethics: they touch upon a number of ethical issues customarily found within that discipline.

## Bioethics

It is easy to forget that for many years ethics, like aesthetics, was a marginalised topic in philosophy. According to Stephen Toulmin [70], the moral philosophy of the first six decades of the twentieth century concerned itself more with locating issues of an ethical nature, rather than with attempting to offer solutions to those issues. Toulmin argued that it was only when it became attached to medicine, in the form of bioethics, that ethics again became a major subject requiring serious consideration.

Bioethics evolved in order to deal with the particular set of ethical issues that arose with the advent of modern biotechnology.^15^ Van Rensselaer Potter [54] coined the term in 1971 to mean an interdisciplinary ethics that would incorporate humanity’s obligations to the total ecosystem. Since then, it has been defined in a number of ways by professionals from different disciplines with divergent interests, so at this point, multiple definitions of this term coexist.^16^ Some theorists delineate it as dealing specifically with the ontological status of the human, or more narrowly with biomedical issues [26]. Others have challenged what they see as a limiting anthropocentric model of bioethics, and wished to include non-human animals in the moral circle [64, 78]. Today, the discipline of bioethics deals first and foremost with regulatory frameworks for the life sciences [72]. Applied bioethics contributes to practical assessment of whether something should or should not be done, on a case-by-case basis.

Bioethicists typically argue according to one of three ethical logics: virtue ethics, deontology (duty ethics) or consequentialist ethics. By far the most common in institutionalised settings, especially in the USA, is consequentialist arguments, in particular utilitarianism. In Europe, bioethicists have tended more towards virtue ethics, which supposes that an action is good if it is performed for the right reasons, grounded in the notion of the common good. Deontology, the belief that universal principles, not the consequences of your actions, determine what is right, is not uncommon in religious bioethics and is also sometimes presented in philosophical perspectives on bioethics, but rarely to be found in applied, institutionalised settings [30, 78].

As a philosophical tradition, bioethics also treats a broader range of ethical questions, such as how our society should relate to the patenting of human genes and gene therapy, the possibility of neo-eugenics as a result of the technology to “purify” embryos, issues of cloning, the use of animals as organ donors and genetic manipulation in agriculture [24, 56]. Since the late 1990s, bioart has been discussed within this wide definition of bioethics. However, upon mentioning “bioethics”, few writers, whether scholars or artists, have gone on to define their understanding of the term. One might speculate whether, in some cases, the very prefix of “bio-” in front of both “art” and “ethics” was the relevant link.

A deontological ethics requires, in Kantian terms, that we do not let the ends validate the means. However, according to consequentialist and virtue ethical perspectives in bioethics, some ends are considered important enough to justify bending or even breaking the existing norms (in some cases, laws) for ethical conduct. Such ends are for instance the curing of major diseases, ensuring food, water and energy supply and other goals that impact on the continued existence and quality of life of a large number of human beings. In such cases, we find justification for the testing of toxins on animals, life manipulation and the use of human stem cells in research, as well as a certain amount of risk. The more massive the problem, the more we will be willing to sacrifice our standards of “do no harm” in favour of the “greater good” (see e.g. [5]).

One of the core philosophical questions of bioethics is: When do we start to consider a cause important enough for such waived standards? Clearly, when there are many human lives at stake. But in most cases, a scientific research project can hold no guarantee that the results will indeed save lives—even when this has been the explicit aim of the research proposal. Furthermore, multiple branches of what we term “scientific” research can hardly be described as being vital to the wellbeing of humankind, and many of these can potentially have the opposite effect. For instance, projects to condition the movements and behaviour of rodents through brain microstimulation [69, 73] and insects through neuromuscular stimulation (see e.g. [60]) mostly have surveillance applications. Are such forms of research more morally permissible than artworks that use laboratory resources, animals or other living materials to create discussion? Frances Stracey [67] poses the question whether biotechnology should even be made available outside the realm of research and industry. What ends justify the use of living materials and expensive equipment? The answer to this question will, inevitably, vary according to one’s ethical framework.

Both bioethics and ethics in art deal with the normative, though with different forms of values. Bioethicist Paul Macneill and art theorist Bronaċ Ferran emphasised in the article “Art and Bioethics: Shifts in Understanding Across *Genres*” [44] that both practices “raise questions about medicine, human composition, and life—but from different perspectives”, and they may complement each other. They also argued that “bioethics itself may be challenged in that answers that rely on common-place formulations such as ‘balancing benefits and harms’ are not so easily applied to aesthetic projects” ([44], p. 80). This is a point mentioned by many scholars discussing bioart (see e.g. [1, 31, 51]).

Macneill and Ferran considered artists to be able to “demonstrably enliven and animate significant topics and themes, including many of interest to bioethics, and develop new forms of engagement that allow for participation and discovery through enactment and embodiment and not just through abstraction or theory” ([44], p. 83). In addition, in contrast “to the consistent seriousness of science, medicine and bioethics, their work can also employ fun, light-hearted or ironic strategies and techniques, although with an equally serious intent” (ibid.). As we shall see, the perceived lack of seriousness has been considered by some as an argument against any use of living materials in art.

### Institutionalised Ethics Meets Bioart

In practice, applied bioethics often takes the form of a committee deciding whether or not a given research project should be allowed to proceed. Important in these decisions is the judgement of whether the perceived gains outweigh the possible harms of a specific project. When artists are formally affiliated with a research institution, as is the case for Oron Catts and Ionat Zurr from the TC&A, they are subject to the same rules that apply for scientific researchers.

Catts and Zurr are based at SymbioticA, the world’s first Centre for Excellence in Biological Arts, situated within the School of Anatomy, Physiology and Human Biology at the University of Western Australia (UWA). When planning a new project, the artists, like any of their biologist colleagues, have to submit their project proposal to an ethics committee. This committee normally consists of medical professionals, perhaps a few biologists and an ethicist or philosopher.

In the first major project conducted by the artists and their collaborators at the UWA in the early 2000s, the ethical committee members were at a loss as to how to relate to a project with ends they were not set up to deal with, and deemed themselves unqualified to assess it. In the end, they decided to “assess the scientific merits of the work initially and then to sponsor and initiate debate on the use of animals for artistic reasons” ([13], p. 134). They intended this as a catalyst for a new type of committee to be convened, in which relevant artistic expertise would also be included. However, 15 years later, the projects at SymbioticA are still evaluated by the same ethical committees. Zurr and Catts [77] argue that this is unfortunate for artists, as the committees tend to focus on whether there is a recognisable method and rigor to the project (there often is not, as artistic research can proceed along quite different paths). On the other hand, as some of the biologists connected to SymbioticA as well as the artists themselves pointed out in my interviews with them,^17^ the process of applying for ethical clearance might help raise the artist’s awareness of potential dangers, ethical issues and other aspects of their proposed project. Artist Anna Dumitriu and ethicist Bobbie Farsides’ edited book *Trust Me, I’m an Artist* shows that many artists outside of SymbioticA, too, have been frustrated with the demands of the system. However, the editors argue that “[e]ven when artists fit into science research groups well and seem to ‘play the game’, their work can raise novel ethical issues specifically because they have become embedded within scientific institutions” ([22], p. 5).

## Bioethics for Bioart, as Seen Through the Prism of the Ethical Criticism of Art

Discussions of what is at stake in bioartworks tend to focus on questions such as: Should artists be allowed to meddle with life? What are the potential implications of artists letting laboratory life forms into the environment? Should there be constraints on whether, how and when artists can use these biotechnologies? (see e.g. [43, 67]). These questions are, importantly, *art-specific*. The ambiguity of art is a common topic in the context of bioart. Artist and writer Ellen K. Levy [43], in her discussion of Eduardo Kac’s *GFP Bunny* (2000, Fig. 3), poses the question of how much factual information should be expected from an artwork. *GFP Bunny* revolved around presenting a transgenic, glowing green rabbit to the audience, but the story presented by the artist was met by a counter-story from the scientist with whom the artist claimed to have collaborated [34]. Their French lab did indeed produce rabbits modified with green fluorescent protein (GFP), but they did not glow the uniform green of the image Kac presented. What ethical implications can there be if the rabbit as Kac presented it, as a creature specifically designed for his art context, didn’t exist? Levy argues that this specific ambiguity is, in fact, an ethical problem, and notes that, “an artist may be encouraging others to perform genetic manipulations that he, himself, has neither commissioned nor undertaken” ([43], p. 203). Her caution is based on a (Platonistic) moralist acknowledgement of the harm that art can do, in this case that members of the audience may be inspired to do something that the artist claims to have done (but probably did not do).

**Fig. 3:**
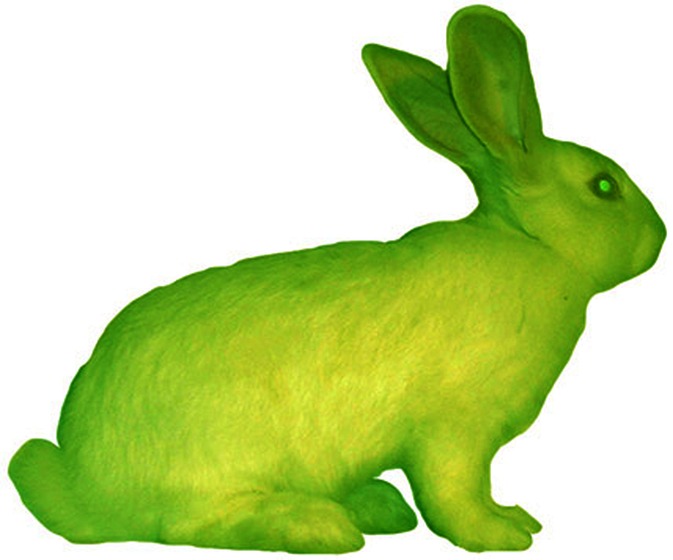
Eduardo Kac, *GFP Bunny*, 2000. Photo credits: Chrystelle Fontaine

On the other hand, this very ambiguity may also spur ethical reflection in viewers. Compared to artworks presenting explicitly fictional modified creatures, such as Vincent Fournier’s *Post Natural History* (2012), a series of photographic speculations about “upcoming species” inspired by synthetic biology and cybernetics (including such creatures as “*Oryctolagus cognitivus*”, a very intelligent rabbit, and the “*Buccus magnetica*”, a goat with the ability to control and generate electromagnetic fields),^18^ the claim of *realness* of Kac’s green bunny seems to have inspired much more media attention, provocation and also reflection. *GFP Bunny* did bring the idea of GFP modification, a common procedure in labs around the world, to a new audience.

Is it acceptable to create ethically dubious artworks, if they cause an increase in reflexivity and ethical awareness of the issues in question? Art critic and researcher Annick Bureaud defined bioart as “an art of belief”, since verification of the artists’ claims, in a scientific paradigm, would require repetition of their processes in the same conditions. She stated that since “this is impossible, we have to ‘believe’ that it is what they say it is—or in some cases, have our ‘doubts’, given what we know to be ‘possible’. Grasping these works takes knowledge, but then shouldn’t a citizen be informed?” [8].

Should we expect a greater degree of documentation, replicability, security and legitimacy from bioart than from art using other media? Art historian Frances Stracey, in an article in *Nature Reviews Molecular Cell Biology*, asserted that bioart is “least successful, and most contentious, when the science is reduced to mere aesthetic spectacle, and no account is taken of the specific or paradigmatic differences that affect how one discipline is mediated through another” ([67], p. 500). At its best, however, she argued that these artworks can be “a provocative reminder that how life is modelled and represented matters to how it is valued, used and disposed of” (ibid.). The view expressed here is implicitly moralist and anti-formalist, seeing a potential in art to foster ethical reflection. Stracey gave two examples of bioart’s best: Critical Art Ensemble’s *Immolation* (2008), which reproduced the effects of incendiary weapons on skin cells in vitro, juxtaposed with large-screen war footage, and Natalie Jeremijenko’s *OneTree* (1998–2001), in which she grew 1000 clones from the same walnut tree, and showed how they developed differently as they were planted in different environments (as pointed out by Jaqueline Stevens [66], due to random factors, even pairs of cloned trees planted in the same environment were not identical). However, Yves Michaud has argued that Jeremijenko’s project also “clears cloning of suspicion and trivializes genetic manipulation” ([45], p. 393). Jeremijenko originally wanted to plant all thousand of the trees, in pairs, at different locations, but had trouble finding suitable sites. Ellyn Shea [63] in a blog entry noted that the 13 pairs of trees that were planted in the San Francisco area have been left without supervision, and that living trees remained at 9 of the sites. This abandoned “afterlife” of the trees shows a difference between flora and fauna: a similar abandonment of animals would not be so easily accepted.

Stracey also emphasised that scientists should communicate more about their role in these projects. Yetisen et al. have recently argued that for scientists, while “the criticism and application of science will undoubtedly continue, perhaps a more profoundly important and yet less recognized contribution may be the ability of bioart to help science understand itself” ([74], p. 732). The scientific advisor at SymbioticA, Stuart Hodgetts, has stressed how “the way I think about my work has actually changed” due to art projects he has contributed to, in particular interactions with artists who consider laboratory processes from a different angle. While he held that his exposure to these artists has given him a greater appreciation of the everyday creative processes in the lab, he also described how he and others who helped the artists at SymbioticA were “frowned upon by their peers”, who saw it as “wasting their time, your time and resources”.^19^

Lawyer Lori Andrews is one of the few who has explicitly engaged with the question: “Should life science artists be held to higher, the same, lesser, or different standards than scientists?” ([1], p. 139). She contends that artists are “generally held to higher standards than scientists” (ibid.), and refers to the example of artist Anthony Noel-Kelly. In 1998, after sneaking away cadaver parts from the Royal College of Surgeons, Noel-Kelly became the first British citizen to be convicted for theft of human remains. As part of the litigation, the RCS received the moulds and casts Noel-Kelly had made of the body parts, to be thereafter included in their anatomical exhibit. Andrews suggests that the approach of “treating artists more harshly than scientists or doctors is suspect” ([1], p. 141). She posits that artworks can “explain to us how biotechnologies work”, and also “provide us with the chance to ask: “What do we want out of our biotechnology?”” ([1], p. 142). At the same time, she stresses the difference in approaches of artists dealing with biology, pointing to Hunter O’Reilly’s painting *Madonna con Clone* as “seemingly intended […] to promote cloning”, whereas TC&A’s *Pig Wings* is presented as an example of artworks aiming to “critically assess the technologies or criticize the manner in which they are being integrated into society” ([1], p. 127). This view of bioart as a form of *manifest vision* is an example of ethical pluralism, in a moderate moralist version resembling Noel Carroll’s perspective [10]. Andrews suggests that artworks’ function of allowing us to critically relate to issues around biotechnology is important and can also “serve as a guide to public policy” ([1], p. 142), by pointing out gaps in existing regulations and potential societal harm from technologies. She stresses that there should also be some legal regulation to prevent artists from crossing boundaries such as making “not a rabbit but a human glow green, or […] to genetically profile a person without consent” (ibid.). This being in place, she argues, bioart can be used “to think about the ways in which people can control the technology, rather than the technology controlling the people” (ibid.).

Andrews, with Joan Abrahamson ([2], p. 1) has also argued, based on a review of “hundreds of novels, short stories, representational artworks inspired by genetics and ‘wet works’ […] that artists, even more than scientists, can make a contribution to the policy surrounding the life sciences”. This research suggests that art can influence the governance of science and technology, as well as affect scientists’ perception of their field. Geneticist Philip R. Reilly expressed the same idea when he described his first encounter with Salvador Dalí’s 1963 painting *Galacidalacidesoxyribonucleicacid*, a large-scale painting featuring a crowd of humans holding hands that form a double helix shape, as the first time he “seriously thought about DNA” ([57], p. xii). Reilly suggested that this experience from when he was an undergraduate student invoked his abiding interest in the exploration of DNA in later years.

On the other hand, a range of writers emphasise that the value of this type of art should not be judged in terms of scientific gains, or even its capacity for making us rethink the technologies in question (see e.g. [31, 46]). Human geographer Deborah Dixon ([21] and media scholar and artist Maciej Ożóg have argued in similar veins that the ambiguous, “liminal ontic status” of the TC&A artworks serves to make us “think about categories such as the body, individuality, identity, specific differences and, last but not least, life itself” ([51], p. 39).

The artworks’ critical potential and aesthetic value are seen as interconnected in both of these accounts, in a positive moralist interpretation of the artworks as conveying a moral critique of biotechnologies, but also potentially a deeper ontological understanding of life (similar views are found in [4, 59, 78, 79]). Dixon argues that whereas the work of Critical Art Ensemble, according to a range of commentators, “is unquestionably political” since “it exposes the realities of global capitalism and seeks to resist the same”, it is a “much more contentious issue” whether more ambiguous work such as that by TC&A is also defined as political ([21], p. 416). Dixon suggests that it is, using the extended meaning of “political” presented by Jacques Rancière. In my contextualist perspective, these considerations are not mutually exclusive.

Media scholar Carol Gigliotti, on the other hand, has taken issue with the “absurdity” of the simultaneous wish to make “humans part of a broader continuum” and the TC&A’s manipulation of life forms which, she claims, “will most certainly not contribute to that project, but only serve to reinforce it” ([29], p. 26).^20^ Gigliotti’s moralist argument touches on important issues of the difficulty of escaping anthropocentrism, and her questioning of both TC&A and particularly Eduardo Kac’s claims to anti-anthropocentrism has value. However, her essay contains a number of odd statements using terms such as “transgenic” and “biogenetics” inaccurately and referring to Catts and Zurr’s work with tissue cultures as “genetic” art. The artists, in turn, have criticized Gigliotti’s “misunderstanding or sloppy use of terms” ([16], p. 129). They describe her approach as an example of a problematic monodimensional social science approach which, contrary to Gigliotti’s explicit intensions, in the artists’ opinion promotes “a reductionist view that manipulation of life through modern biology happens only at the molecular (genetic) level)” ([16], p. 127). Moreover, they argue that artists’ extended “wet” experience in laboratories can be seen as “a political act that goes beyond the democratization of the technology, to the act of breaking down dominant discourses, dogmas, and metaphors to reveal new understands of life and the power structure it operates within” ([16], p. 140). They posit that bioart such as their own, “that deals with other nongenetic forms of manipulation can be used as a way to counterbalance the view of life as determined solely by the DNA code” ([16], p. 129), thus countering the genohype [33] perpetuated by many social scientists. However, the ambiguity of their work means that it can also be interpreted as having quite different meanings than the ones they state in their papers [21]. Also, as Catts and Zurr acknowledge, there can be little doubt that many artists who are working with genetics do serve to bolster genohype, through their interest in DNA as a source of identity (see e.g. [1, 66]).

The range of new ethical challenges in bioart, compared to “traditional” art media, naturally entails the emergence of new questions, and the introduction of new discussions. So far, however, criticisms of bioart have often dealt more with the artists’ intentions and reasonings than with the artworks themselves. While this is understandable, considering that it offers clearer statements to argue against, it disregards the idea that the artworks might well contain issues unthought of by their makers. Performance and media scholar Kate Rossmanith has argued that Catts and Zurr, in their writings, “sell their work short, for the projects don’t only work at the level of ideas. Human cells grown into living, growing sculptures: at stake here is not merely an *idea* or a *representation of life*, but *our experience of being-and-having a body*” [59].

New media scholar Joanna Zylinska [78, 79] has suggested that the commonly posed questions, e.g. of artists’ right to manipulate life, while valid, are not “*the best questions* we can ask about bioart, for the simple reason that they evoke a normative position on life” ([79], p. 192). In her opinion, bioartistic projects can serve to “challenge the traditional humanist value-based ethics, where this nebulous entity called ‘human life’ is posited as a value in advance” ([79], p. 194). She argues that bioart has the potential to participate in “the performative enactment of life as such” ([79], p. 194), materially exploring questions such as “what is life?”, “what is the meaning of life?” and “how do organisms relate to each other?” Some TC&A projects, particularly their feeding and killing rituals, display such performativity. It is important to realise, however, that the artworks themselves, while perhaps raising certain questions in the minds of beholders, leave it up to the audience to make up their own minds about the answers.

Zylinska stresses that bioartworks are not *necessary* to explore such questions, stating that biotechnology is perfectly capable of posing such questions from within, and I might add that philosophers of science, bioethicists and other professionals are also contributing to this discussion. Notwithstanding, “bioart is uniquely placed to undertake this kind of questioning knowingly and purposefully, since it lacks the pragmatic imperative of many science and technology projects, whereby innovation and economic growth frequently overshadow any non-goal oriented agendas” ([79], p. 194). Zylinska argues that artists do take responsibility for life and that they are materially engaged in “enacting a different ethics of life” ([79], p. 195). This view, while contextualist in the sense of stressing extra-aesthetic properties of the artworks, stretches the framework of ethical criticism of art in arguing that the most interesting questions to pose in relation to bioart are ontological in nature.

Zylinska does acknowledge that many bioartworks do not live up to the possibilities that she sees for the genre, that it is perhaps rare to find the transformative responsibility for life that she considers its full potential. This is an important point: not all bioartists have the same view of their own ethical responsibilities, and they have very different boundaries for what they will and will not do. George Gessert, one of the “grandfathers” of bioart, has worked with plants for decades and explains his choice among other reasons with there being less serious ethical considerations in working with them: “The prerequisite for conscious experience is a nervous system, which plants lack. This makes them, along with fungi, microorganisms, and cells in vitro, invaluable materials for artists” ([28], p. 22). He specifies that, although there are still ethical considerations, they are not as severe as in working with mammals. Catts and Zurr, although working with cells, named in Gessert’s list of “invaluable materials”, in turn refer to a sense of *discomfort* as an important factor in their work: they state that they want to work with technologies they are uneasy with, and seek to spread that unease.^21^

Philosophers Thomas Brian Mooney and Samantha Minett, on the other hand, argue in “If pigs could fly, should they?”^22^ that art is not sufficiently serious a cause for doing any kind of harm: “aesthetic appreciation may appear frivolous when calculated against animal suffering” ([47], p. 632). In their view, the potential benefits of science may weigh heavier than concern about animal welfare, while art cannot offer similar benefits. They posit that the use of animals for art is morally suspect, and therefore, all use of animal-derived cells or DNA is also problematic [47]. However, most ethicists, regardless of their moral philosophical framework, will agree that there is a difference in kind as to our responsibilities to single cells and higher mammals.^23^ If we take the common decisive factor of whether or not the organism involved is capable of feeling pain, cells without a neural network connected to it would be excluded from moral consideration. The ethical issue would concern the inability of the animal to consent to donating the cell.

The TC&A, when growing, for instance, rat skeletal muscle in vitro, consider themselves “scavengers”: they obtain starter tissue from scientific researchers and do not biopsy the animals themselves to get the tissue.^24^ As such, their responsibility rests in the first instance at the cell level, since the animal’s tissue was originally harvested for science, and the cells cultivated from it exist independently of its originator. More problematical is the use of foetal bovine serum (FBS) as the most effective growth supplement (although alternatives do exist, see e.g. [35]) for tissue culturing of eukaryotic cells. FBS is a by-product of the meat industry, produced from the blood of foetal calves taken from the wombs of butchered cows. As long as FBS is used as a nutrient for the cells, the resulting products will not be *victimless*. Catts and Zurr estimate that “growing around 10 grams of tissue will require serum from a whole calf (500 ml.), which is killed solely for the purpose of producing the serum” ([16], p. 133).^25^

The TC&A’s use of FBS does invite the question of whether the use of biotechnological animal products in art is morally defensible. If one takes a moralist outlook, this may be seen as a devaluing factor for the artworks. However, “translating” to a more traditional artistic medium, this would also apply to art supplies produced by child labourers, and paints that cause harm to the environment. Risks caused by exposure to volatile organic compounds in producing, handling or interacting with artworks would arguably fall into the same category, but this is rarely mentioned in moralist assessments of paintings. The ethical discussions of bioart can thus also point to limitations in the ethics of art: its theoretical bird’s eye view rarely takes the process of production into account when judging an artwork, and even moralists relate to the artwork as autonomous in the sense that it is the content of the artwork itself that is judged as (im)moral.

### Alternatives Instead of Living Art?

Comparative literature scholar Krzysztof Ziarek ([76], p. 95–96), discussing *GFP Bunny*, has questioned “whether art is actually needed in order to generate the kind of discussion, no doubt crucial and imperative, that has been going on around Kac’s work, or whether those questions do not in fact arise from the very premises, objectives, and capabilities of genetic technology”. Although in some cases, such as genetic privacy and human cloning, this will clearly be the case, many emerging technologies and projects go very much “under the radar” in the public sphere. Despite important research conducted in recent years in the fields of public engagement and science communication, what is ordinarily being communicated from scientific research is still the *result*, not the process of research and the means employed. Debates are to a large extent carried out within the research fields, where the parties are informed on, mostly also have interests in, the issues in question. Ethicists may be invited in to provide their “expert opinion” in the discussion, but real public debate on these matters is rarely seen, partly due to the technical language often used in scientific discourse. There appears to be little doubt that the affective, material connection that art offers can involve new groups in the discussion. Is this a sufficient justification for it within a moralist and/or utilitarian framework?

**Fig. 4:**
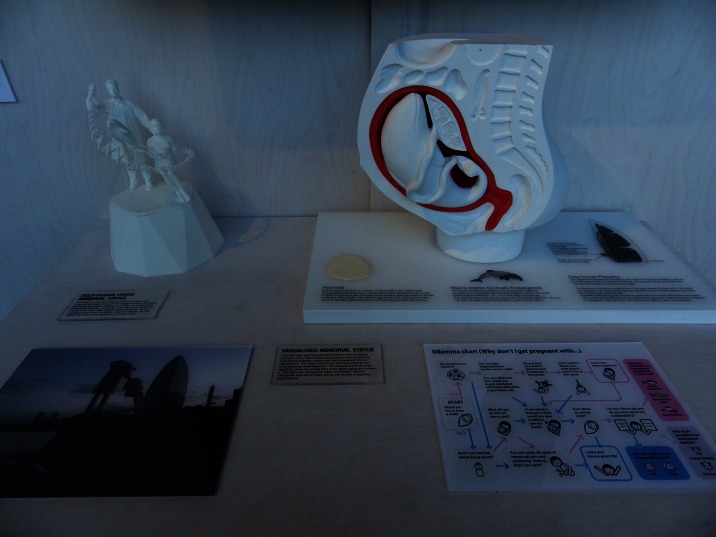
Ai Hasegawa, *I Wanna Deliver a Dolphin*, 2011. Exhibition photo from Grow Your Own… Life after Nature, Science Gallery Dublin. Photo credits: Nora S. Vaage. Reproduced with permission from the Science Gallery

The ethical, societal and cultural issues of biotechnology have been dealt with by a number of artists using “non-wet” media such as painting (Alexis Rockman) or photography (Vincent Fournier) rather than the methods of biotechnology itself.^26^ Ai Hasegawa, in the speculative design piece *I Wanna Deliver a Dolphin* (2011), presented a scenario where human beings with adapted placentas could give birth to endangered dolphin species. Using an “anatomical section” sculpture of the human womb containing the dolphin foetus, pictures of a “dolph-human” future, and a video of herself “giving birth” to a dolphin in a swimming pool, Hasegawa richly explored the potential of such a technological future using “traditional” media (Fig. 4).^27^ In addition, of course, bioethicists, philosophers of science and other academics treat the same issues through verbal arguments.

So, does the existence of these less ethically problematic alternatives add to the argument that it is morally indefensible to use bioscientific techniques for the “frivolous” purpose of art (cf. [47])? Hasegawa is a graduate of the Royal College of Art’s Design Interactions programme, in which Anthony Dunne and Fiona Raby have in the past two decades developed the approach of “speculative design”, which they describe as being “about meaning and culture, about adding to what life could be, challenging what it is, and providing alternatives that loosen the ties reality has on our ability to dream” ([23], p. 189). Catts and Zurr will, in fact, for the year of 2016 be visiting faculty at RCA, where Dunne and Raby stepped down as faculty in 2015. Since their appointment last year, Catts and Zurr, who have previously stressed that the license that they have to make their works is also founded in their status as art, have started defining their practice as “contestable design” [55], suggesting that the boundaries between art and design, as well, are at present quite malleable. These approaches are, then, not only similar, but interlinked.

The “semi-living” artworks from the TC&A are striking in their very live-ness. Oron Catts, in “Not Moving—Living” [12], relates a long process of making their immobile cell creations appear *visibly* alive. Simply being *told* that the artworks were alive did not have the same affective force. The dampness inside the bioreactors, the shrunken remains of the *Pig Wings*: these artworks confront us with life that has been created, in some cases connecting the experience directly to the idea of “what if this was for my consumption?” If the artworks were not *alive*, grown through the method of mammalian tissue culturing, those implications would be lost. Concurrently English scholar Robert Mitchell argues about TC&A’s *Disembodied Cuisine*^28^ that this effect is valid also when the actual, living artworks are absent, as “even simply learning that such a project is “out there somewhere” can produce a sort of adrenalized, excited concern (or even crisis) on the part of some who read or hear about this project” ([46], p. 72).

In a sense, bioartists can get closer to biotechnology than can philosophers of science, as they can *demonstrate* biotechnology by producing their artworks. On the other hand, the materially manifest nature of these artworks can itself generate an ethical problem, as we have seen. Philosopher of technoscience Alfred Nordmann has recently pointed out how “[o]ne can be prepared for the future without seeking to know what the future will be like” ([49], p. 88). Instead of striving to imagine all possible or plausible futures, he argues, we might start out by observing our present situation. This is precisely what the TC&A seem to be doing: materialising possibilities in our present that simultaneously carry visions for the future and show our technical and ethical limitations. Through materialising technological utopia ad absurdum, sardonically showing what could be achieved with current technology, the TC&A are both performing and critiquing potential usages of the technology [28]. We might consider these artworks as a type of material technology assessment, with an expanded license to speculate. As discussed above in relation to Hasegawa, Dunne and Raby, such speculation can be manifested through prototypes that are not, in fact, alive, still giving room for visions of the future. However, the presence or absence of a *living* element does appear to make a difference.

As pointed out in the introduction to this paper, any engagement with the ethics of bioart begs the question: what does this art *do*? What difference does it make? The effect or impact of an artwork is notoriously difficult to assess. However, the potential impact might be suggested through looking at some of the properties of the artwork: visceral, alive, *other* [46]. Not to be touched, and when it is, fragile to the point of inevitable deterioration. The audience’s role as contaminators of the cells in the TC&A’s killing ceremonies may give rise to reflections around the status of different classes of organisms. It may make some of us more aware of the everyday world around us. In the case of a moralist response, that very viscerality is cause for concern.

Complete autonomism makes little sense in the case of bioart, where concept tends to be at least as important as form (see e.g. [46]). The idea of aesthetic autonomy does not cover the critical and narrative potential of biological artworks, and I have not found a single example of a scholarly autonomist critique of bioart. However, the idea of artistic licence, artists being allowed to do things that would not be permissible in other contexts “for the sake of art”, remains a frame of reference. Oron Catts, although now starting to describe himself as a “contestable designer” [55], has previously often commented on how defining himself as an artist and the work as art “is the best place I can position it” as “it gives me a license to do things in ways which are very different than any other profession”.^29^ This idea also importantly shapes the TC&A’s views of how one should, and should not, engage politically and innovatively through artworks. Catts objects, for instance, to ecological art presented as “one of the tools to solve the problem”, stating that it is “betraying the license that we have as artists […] this instrumentalisation of art, regardless if it’s for a good cause or a bad cause, it’s a problematic way of positioning art”.^30^ Apart from this notable inheritance from an autonomist view on art, we can glean from the ethics of art framework the extent to which different commentaries on bioart are moralist or contextualist, and how this affects their view not just of the artworks, but also of the topics they treat.

Any artwork made using biotechnology engages, explicitly or not, with the societal context of the biotechnosciences. Whether the artwork critiques current procedures, poses questions concerning future applications of a technology or utilises the available techniques as a medium to pursue aesthetic goals, it cannot be said to have intrinsic value only. Although, as artworks, their aesthetic expressions are part of what move us, the societal context, the ethical implications and the technological possibilities all play a role in the fascination this type of art holds for many of us. Zylinska ([78], p. 156) has suggested that “the tactical effects of many bioart projects lie in their ability to shift the discourse on, and of, (bio)ethics”. Artist Boo Chapple [19], building on the 1917 essay “Art as Technique” by Russian formalist Viktor Shklovsky, has suggested that an important property of bioart is its capacity to “make strange” our familiar representations of the world, in particular the laboratory and the biotechnological object. This *making strange*, I suggest, can then be a factor in *making sense* of the object or idea under scrutiny.

Can art contribute to moving boundaries, in individuals or in society at large, as to what is ethically acceptable? And if so, in what direction? It may well be that artworks that stretch our moral perception can indeed contribute to our more speedy acceptance of new technologies and their potential usage. As Andrews has pointed out, some of these artworks may “lead to greater acceptance of biotechnology, because it makes it seem like the technology is attractive, safe, or valued” ([1], p. 127). Economic and social theorist Jeremy Rifkin has argued that this kind of art is likely to “legitimise the idea of a new ‘artful’ eugenics movement” [58]. He sees it as mirroring a tendency for spectacular science, in such projects as the Vacanti mouse with an ear on its back, or goats producing silk in their milk. The TC&A, as well as Kac and Critical Art Ensemble, are specifically mentioned as contributing to this trend. However, Catts and Zurr share Rifkin’s distrust of spectacular science and have repeatedly discussed how their artworks ironically engage with the hype resulting from such spectacles [14, 17]. Ożóg has pointed to the TC&A’s endeavours to get the audience to engage with the semi-living sculptures, precisely because “due to their small size and the fact that they grow very slowly”, they “are not spectacular in character” ([51], p. 45). However, within their ironic approach, the artists do play upon the spectacular.^31^

## Concluding Remarks

A fundamental ethical question, touching upon many aspects of life, is: “Should we do things just because we can?” Works of art, I argue, can contribute to an answer by providing a materialised visualisation of the issue at hand, a demonstration with different connotations and aims than those of research, and invoking different faculties (affect, emotion, reflection) in the reception. At the same time, that very question is often asked *in the context of these artworks*, precisely because the artists and their collaborators are also “tampering” with nature. There does seem to be a lot at stake here. Bioartworks, and commentaries from the audience, can play a role in widening or tightening the fields of possibility that artists are trying to create awareness of, thus potentially influencing future decisions as to what our society should be like. As such, discussions raised by these art pieces are closely interconnected with those of technology assessment and philosophy of technoscience.

If people are confronted in an embodied way with something they would not have thought of themselves, it may spur them on in developing their personal ethical framework [43]. Several artists and hackers working with biology have stressed that often, participants in interactive bioart workshops might start out with an attitude of “let’s do this”, and only gradually become aware of ethical issues inherent in what they are actually doing.^32^ Exposure, then, can also lead to contemplation of the issues at stake.

In a contextualist view, the TC&A artworks’ ethical dimensions, including the problematic use of FBS, add significantly to their ability to affect and induce such reflection. These properties are vital to the artworks’ capacity for raising discussion around current ethical problems, questioning what might otherwise be taken for granted. Whether or not this is considered part of art’s prerogative will depend on one’s ethical framework. Therefore, awareness of how that framework influences one’s opinions can bring increased depth to discussions of the ethics of bioart and its relationship with biotechnologies.

## Footnotes

1 The anthology *Signs of Life: Bio Art and Beyond* [40] has a section on “bioethics”, including a chapter by bioethicist Cary Wolfe, and another anthology on the topic, entitled *Tactical Biopolitics: Art, Activism, and Technoscience* [20] includes chapters categorised under the headline “biosecurity and bioethics”, notably one by Paul Rabinow and Gaymon Bennett.

2 Levy discusses “ethical issues” as such and does not refer to the term bioethics, or to any other ethical framework, in this article.

3 N. S. Vaage forthcoming: Fringe Biotechnology: DIYbio, Art, and Other Approaches at the Institutional Outskirts.

4 As has been pointed out repeatedly in recent years (see, e.g. [50]), art theorists are becoming less concerned with definitions of art and more with its *functions*, its *effects*—precisely this question of what art can *do*.

5 This article is focused on a small range of “living” artworks created using biotechnology. However, the category of “bioart” is quite heterogeneous, and many scholars include such different activities as genetic portraits (Marc Quinn, Iñigo Manglano-Ovalle) and bacterial paintings (David Kremer), interactive do-it-yourself workshops (Reiner Maria Matysik, SymbioticA, the Waag Society), bioelectronics (Hackteria) and certain types of body art (Stelarc, Orlan, Art Orienté Objet) in the term (see, e.g. [31, 46]).

6 The case study, performed in February–May 2013, included semi-structured interviews with the involved artists and biologists, in addition to participant observation, archival studies and material study of documentation of the artworks.

7 I analyse the academic reception of bioart, in which ethical issues have been discussed at some length. The same approach could well be used, for instance, to analyse audience or media reception.

8 Personal conversation with Oron Catts, April 2013.

9 In recent years, the artists have focused more on the origins of life, “the substrate”, and the historical background of the engineering approach to biology in pieces such as *Crude Matter* (2012) and, with Corrie van Sice, *The Mechanism of Life—After Stéphane Leduc* (2013).

10 The use of the term “art” when discussing the ancient Greeks is, of course, an anachronism, as their concepts of *techne* and *poiesis* did not carry the same connotations as our contemporary conception of art.

11 What I here term “autonomism” is sometimes referred to as “formalism” (a term that also carries other meanings), see, e.g. [37]. Similarly, in recent years, the term “ethicism” has partly taken over for the term moralism, see, e.g. [27].

12 Consider the conflict between the proponents of *disegno* (drawing/form) and colour in the Italian renaissance.

13 “Art for art’s sake”, a phrase attributed to the early nineteenth century poet Théophile Gautier, today alludes both to the aesthetic autonomy of an artwork, suggesting the ideal of pleasure without interest and no interpretation outside the frames of the picture, and to the institutional autonomy of the art world.

14 The explication of these positions must be viewed as a theoretical tool. In real life, a pure immoralist, like a pure moralist or autonomist, is very rarely to be found.

15 Engelhardt ([26], p. 95) also argues that the discipline “came into existence to fill the moral and cultural vacuum that resulted form the deprofessionalization of medicine and the secularization of the West”.

16 Within the genomic sciences, another term sometimes preferred is “genethics” [68], and “neuroethics” [53] has also been proposed for the neurosciences.

17 Research interviews at SymbioticA, April–May 2013: interviewee 27; 42; 44; 49; 53; 56. Interviewee 28, an artist in residence, on the other hand, referred to the process as “a joke”, there “to make a broader public feel better about what’s going on”. The interviewee did add that ethical clearance “does have some protective boundaries”, but stressed that it “is not about ideas. I don’t feel like the ethics department here is interested in what is ethics per se”.

18 Fournier’s piece was exhibited at the *Grow Your Own… Life After Nature* exhibition at Science Gallery Dublin, 2013–2014.

19 Research interview with Stuart Hodgetts, SymbioticA, May 2013.

20 Similarly, N. Katherine Hayles asks in the context of Eduardo Kac’s transgenic bacterial artwork Genesis: “Does Kac’s intervention in the genetic sequences of bacteria contest the notion that humans have dominion or reinforce it? The ambiguity inheres in any artistic practice that uses the tool of the master to gain perspective on the master’s house” ([32], p. 83).

21 Research interviews with the artists at SymbioticA, UWA, April–May 2013.

22 The title is an explicit reference to Catts, Zurr and Ben-Ary’s *Pig Wings* (2001).

23 An exception would be the abortion issue, in which some would argue that even the smallest embryo’s potential to become a human being entitles it to be afforded already the rights of a human being.

24 Research interview with Ionat Zurr at SymbioticA, May 2013.

25 This is not necessarily accurate: FBS is mainly produced in areas where cattle roam free, and an entire herd is taken to slaughter at the same time, including some pregnant individuals. The foetuses, then, are not killed directly for their serum. However, the process in which the serum is extracted may take more than 30 min, and may quite possibly be painful to the neonates [38].

26 Mitchell [46] has introduced the distinction between “prophylactic” and “vitalist” bioart, in which the prophylactic tactic involves insulating the art and its spectators from the problematic of biotechnology, critiquing from a distance. Rockman, in particular, is used as an example of this tactic. On the other hand, the vitalist tactic involves engagement with biotech from within.

27 The piece was exhibited at the *Grow Your Own… Life After Nature* exhibition at Science Gallery Dublin.

28 In this piece, TC&A grew skeletal frog muscle cells into little steaks and served them to eight selected diners at the L’Art Biotech in Nantes, 2003. Mitchell is also referring to Beatriz da Costa and Critical Art Ensemble’s *Transgenic Bacteria Release Machine*, part of the *GenTerra* project.

29 Catts has a degree in product design, and his partner Zurr in media photography, so their framing of their practice as art, at the time they started tissue culturing, represented a conscious act of self-positioning. Since then, Zurr has earned a PhD in Art Theory and History, and Catts has a Master’s in Visual Arts. Research interview with Oron Catts at SymbioticA, April 2013.

30 Research interview with Oron Catts at SymbioticA, April 2013.

31 Research interviews with the artists at SymbioticA, April–May 2013.

32 [75] and personal conversation with Marc Dusseiller of Hackteria, Nov 2014.
